# Strategies and limitations of the bat immune response to *Pseudogymnoascus destructans*: the causative agent of white-nose syndrome

**DOI:** 10.3389/fimmu.2025.1736823

**Published:** 2026-01-12

**Authors:** Maya J. Jacewicz, Noah P. Rogozynski, Brian Dixon

**Affiliations:** Department of Biology, University of Waterloo, Waterloo, ON, Canada

**Keywords:** adaptive immunity, bats, Chiroptera, innate immunity, Pseudogymnoascus destructans, torpor, white-nose syndrome

## Abstract

The rapid spread of white-nose syndrome (WNS), an invasive fungal pathogen in bats caused by the psychrophilic fungus *Pseudogymnoascus destructans*, represents one of the most severe ongoing wildlife disease crises in North America. Since its emergence in 2006, WNS has driven drastic population declines in several ecologically and economically important bat species, including *Myotis lucifugus*. Once widespread and abundant, *M. lucifugus* is now designated as vulnerable or endangered in several jurisdictions, such as under Ontario’s *Species at Risk Act* (SARA). Despite this, current gaps in understanding the host-pathogen interactions underlying WNS have created uncertainty about which physiological or immunological pathways should be targeted for potential mitigation strategies. The infection produces lesions on the wing and tail membranes of the host, leading to excessive arousals from hibernation and premature depletion of overwinter fat reserves. Early defense begins on the skin, with inhibitory microbiota and acidic conditions assisting in the prevention of fungal germination. Upon infection, fungal antigens are recognized by pattern recognition receptors including toll-like receptors (TLRs) and C-type lectin receptors (CLRs), which trigger a cascade of cytokines that elicit the acute phase response (APR). This process typically initiates recruitment of innate immune cells for fungal clearance, such as macrophages and neutrophils, although North American bats show limited success in early mobilization of these cells to sites of infection. This failure to respond effectively is likely a result of an over-skewing towards a T-helper (Th)17-type response, identified by upregulation of cytokines such as interleukin (IL)-6, transforming growth factor (TGF)β, and IL-23. In contrast, *P. destructans* incites a Th1-skewed response in vaccine-challenged bats, which proves to be more effective in controlling fungal proliferation and suggests antagonism between the two response phenotypes. Antibody-mediated immunity appears to assist in survival, but is not a primary mechanism for fungal clearance, instead contributing to the prevention of excessive wing lesions. Discerning the immunological differences between susceptible and resistant bat populations is essential for developing effective strategies to mitigate the impact of WNS and may reveal novel insights into the complexity and potentially maladaptive nature of Th17 responses in North American bats.

## Introduction

As key contributors to North American ecosystems, bats support agricultural productivity by pollinating economically significant plants and naturally managing insect populations, diminishing the need for chemical pest control (1–3). For this reason, the rapid decline of hibernating bat populations across Canada and the United States due to white-nose syndrome (WNS), a fungal disease that has caused over 90% population losses in some species since its detection in 2006, is a serious concern with far-reaching ecological and economic implications (4). *Pseudogymnoasus destructans*, the causative agent of this disease, is a filamentous, psychrophilic fungus in the phylum Ascomycota, a group largely comprised plant pathogens, and is the causative agent for WNS (5). The fungus exists in a dormant form as conidia in the winter environments in which bats tend to roost, called hibernacula. The conidia are transferred from both hibernacula walls and from other conspecifics while the bats are in an active, euthermic period of hibernation, known as arousal, and then proceed to germinate on the skin of bats during a state of reduced metabolic activity, or torpor (6). Infection begins when bats come into contact with *P. destructans* conidia, initiating colonization of the skin, characterized by white fungal growth on the face, ears, and wings of infected individuals (7). As infection progresses, fungal filaments, or hyphae, breach further into epidermal tissue, damaging wing and tail membranes and causing lesions that hinder thermoregulation and gas exchange (7). Increased fungal biomass results in inflammation and irritation that disrupt hibernation patterns and deplete fat reserves as the host arouses more frequently (7). Eventually, the host may either succumb to starvation or clear the infection, shedding conidia back into the surrounding environment (5, 7). Interestingly, most other species in this class of fungi are plant pathogens and thus, *P. destructans* exhibits several modes of infection that are often characteristic of this group such is biotrophy, an invasive strategy in which *P. destructans* will retain the viability of the invaded tissue to obtain nutrients (6, 8).

In parts of the United States where bats currently combat WNS, the collective economic loss caused by the agricultural destruction and the subsequent need for chemical pesticides attributed to declining bat populations was estimated to be $26.9 billion between 2006 and 2017, equating to over $35 billion today, when adjusted for inflation (1). Although understanding WNS is essential for developing effective conservation strategies, the immunological responses of bats to this disease remain poorly characterized. Due to the relevance to human public health, current research in bat immunology has predominantly focused on antiviral immunity, resulting in a substantial knowledge gap regarding how bats respond to extracellular pathogens such as fungi (9). Nevertheless, this information may be advantageous in discerning why bats struggle when facing a fungal antigen. This work outlines the current understanding of a typical immune response to *P. destructans*, comparing the variable responses between bat species in Europe, to which the fungus is likely endemic, and the far more susceptible North American species (5). The compilation of this data offers critical insights into antifungal immunity in bats, laying the foundation for advancing research on antifungal immunity in bats and safeguarding the ecological and economic benefits that these animals provide.

## Innate response

### Cutaneous defenses

**Figure 1:**
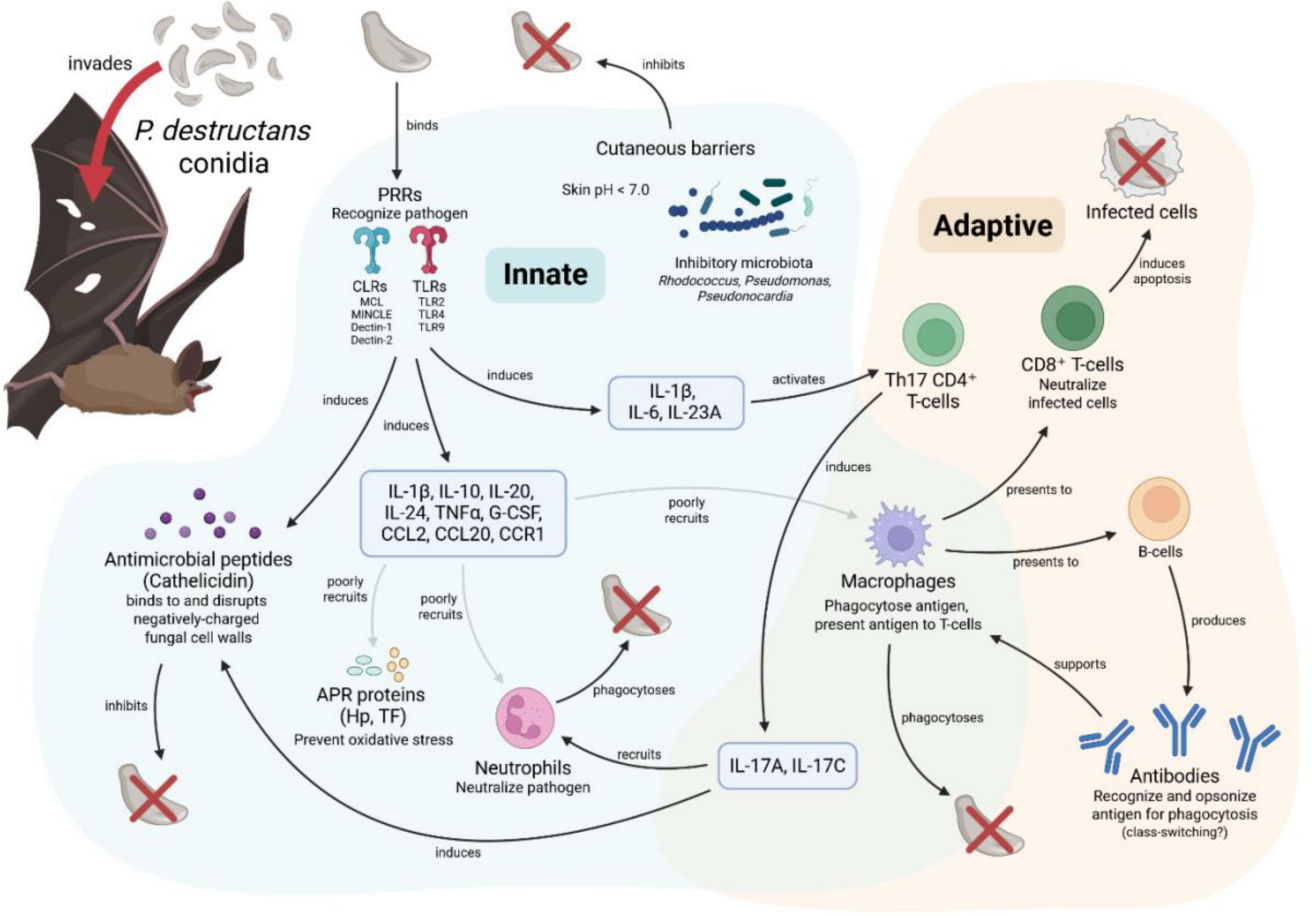
Known mechanisms in the immune response to white-nose syndrome in North American bats.

The ideal temperature range for *P. destructans* growth is between 12 and 16 °C and thus, infection in bats predominantly occurs during hibernation, a period of reduced metabolic rate and body temperature (5). A WNS infection begins with the adhesion of conidia to the epithelial surface of bats and so, an early mechanism of defense against *P. destructans* is the cutaneous microenvironment (5). In the past, higher skin pH levels have been linked to greater vulnerability to skin infections in various mammals, including humans, mice, and dogs, with similar conclusions recently being drawn for bats as well (**Figure 1**; 10–13). To this end, a 2021 study by Vanderwolf et al. measured the average external skin pH from three skin sites of individuals from several North American bat species, including *Myotis lucifugus*, *M. leibii*, *M. septentrionalis*, and *Perimyotis subflavus*, as well as both wild and captive *Eptisecus fuscus* bats, with the five species collectively representing a range of vulnerability to WNS (13). The most acidic skin was detected on *E. fuscus*, which has also been documented to be a species less susceptible to WNS, while *M. septentrionalis*, with the most alkaline skin, has suffered the most severe population declines (4, 13). Although not directly evaluating the relationship between skin alkalinity and susceptibility to the disease, the study demonstrated that skin pH patterns reflected trends in WNS prevalence observed in wild populations (13). With an understanding of species-specific vulnerability to WNS as a consequence of skin pH, targeted therapeutics can be created by altering external host conditions to decrease pathogen internalization (13).

Studies on the composition of external microbial communities in bats suggest a correlation between bacteria found on the skin and in surrounding roosting environments (14–19). Moreover, susceptible North American bat species that hibernate in roosts contaminated with *P. destructans* spores have been found to harbour skin microbiota potentially capable of inhibiting fungal growth (16–18). In 2015, Hoyt et al. swabbed *E. fuscus*, *M. leibii*, *M*. *lucifugus*, and *M*. *sodalist* bats and isolated several strains of *Pseudomonas* bacteria with the ability to prevent fungal growth (16). Following this, two studies conducted in 2017 and 2020 by Lemieux Labonté et al. swabbed wild *M. lucifugus* and *E. fuscus* bats, respectively, and using 16s-based PCR analysis, characterized additional microbial communities present on wing membranes (17, 18). Based on these studies, antifungal bacteria from the phylum Actinobacteria, notably those from the genera *Rhodococcus*, *Pseudomonas*, and *Pseudonocardia*, comprise significant amounts of the skin microbiota in North American bats that cohabitate with *P. destructans* (**Figure 1**; 16–18). While these bacterial taxa have demonstrated *in vitro* inhibition of the fungus, the relatively recent fungal establishment on North American bat skin coupled with the continuous mortalities seen across the sampled species suggest that this mechanism is not adequate in preventing fungal infection entirely (17, 18). Notably, species with a greater susceptibility to WNS, such as *M. lucifugus*, displayed a stark decrease in skin microbial diversity in the presence of *P. destructans*, whereas the skin microbiota of more resistant species, such as *E. fuscus*, remained unaffected when *P. destructans* was present, suggesting deleterious effects of the fungus to the cutaneous microbiome in vulnerable species (14). Despite displaying a similar susceptibility to WNS as *M. lucifigus*, however, *P. subflavus* bats demonstrated a reduction in bacterial microbiomes much like that of *E. fuscus*, implying that understanding impacts on skin microbiota alone cannot fully predict vulnerability to disease (14).

*In vivo* treatments involving the application of *Pseudomonas fluorescens* probiotic bacteria onto *M. lucifugus* bats simultaneously inoculated with *P. destructans* have yielded promising results (20, 21). With the assistance of the *P. destructans*-inhibitory bacteria, bats exhibited a decreased fungal load, fewer invasive tissue lesions, and higher overwinter survival rates (20, 21). Notably, bats that had been given probiotic bacteria prior to pathogenic exposure did not demonstrate similar reductions in pathology, suggesting that probiotic treatment may be a beneficial tool in limiting disease symptoms in populations already in contact with the fungus, but likely cannot prevent future infections in naïve groups (20). Collectively, while the skin microbiota may contribute to defense, it alone is not sufficient in preventing a widespread infection, prompting the need for additional host immune responses. The assistance of non-invasive probiotic treatments such as those conducted with *P. fluorescens*, however, may be effective in mitigating WNS severity in previously exposed populations.

### Antigen internalization

Aside from the effects of the external skin environment, internalization of *P. destructans* may also be hindered by repeated bouts of torpor and arousal. The psychrophilic nature of the fungus prevents growth above approximately 20 °C and thus, early colonization is interrupted upon each return to regular body temperature (5). Nevertheless, once sufficient fungal hyphae have invaded the epidermis, infection can occur regardless of torpor and arousal cycles (5). *P*. *destructans* conidia that are endocytosed by host phagocytic cells remain viable upon arousal due to spore surface coats containing 1, 8-dihydroxynaphthalene (DHN) melanin, which inhibits their destruction by the phagosome and permits continual germination of conidia (5, 6). Alongside biotrophy, parasitic fungi such as *P. destructans* often employ other strategies to prevent triggering host defense responses (22). These may include the formation of specialized structures to avoid recognition by host receptors and to assist in the attachment, penetration, and proliferation of fungal hyphae, although these mechanisms have not been well investigated in WNS infections (5, 22, 23). In human fungal diseases, several characteristic innate immune cells are employed during early stages of infection, including natural killer (NK) cells and professional phagocytic cells, such as neutrophils, macrophages, and dendritic cells (24, 25). These cell types have also been characterized in various bat species, although not all have been confirmed to participate in the WNS response (26). In humans and mice, cutaneous fungal infections, such as those caused by *Candida albicans*, activate pattern recognition receptors (PRRs) from the C-type lectin receptor (CLR) family and the Toll-like receptor (TLR) family (27, 28). In *M. lucifugus* bats infected with *P. destructans*, several similar PRRs were found to be upregulated in wing tissue, including C-type lectin domain (CLEC) family 4 member D (CLEC4D), family 4 member E (CLEC4E), family 7 member A (CLEC7A; Dectin-1), and family 6 member A (CLEC6A; Dectin-2) from the CLR family, as well as TLR2, TLR4, and TLR9 (**Figure 1**; 15, 29). Each of these receptor subtypes bind to similar fungal ligands in humans and mice, suggesting a conserved mechanism of the recognition of fungal molecules between bats and other mammals (27, 29). With this in mind, other antigen-binding factors, such as adhesin proteins, remain uncharacterized in *P. destructans* and should be investigated further, considering the potential for targeted therapeutics as seen in humans (30). Bats also exhibit a characteristic dampened type I and type II interferon response, a pathway that typically prevents the replication of viruses in other hosts (31). Although highly favourable in the context of viral tolerance, this adaptation likely inhibits the coordination and activation of NK cells, macrophages, and other phagocytic immune cells necessary for early fungal clearance, further delaying the preliminary immune response to *P. destructans* (32).

### Acute phase response and immune cell recruitment

Upon recognition of *P. destructans*, a host immune system typically initiates an acute phase response (APR) characterized by rapid leukocytosis, fever, and body mass reduction, and is often associated with sickness behaviours such as decreased appetite and lethargy (33, 34). In humans and other mammals, an APR is a non-specific response, generally triggered by infection, trauma, or tissue damage, and is critical in mounting an early response to physiological disruptions (35). During this response period, *M. lucifugus* bats infected with *P. destructans* display an eight- to 20-fold increase in expression of cathelicidin, a positively-charged antimicrobial peptide that binds and disrupts negatively-charged fungal cell walls (**Figure 1**; **Table 1**; 38). The complement system, a central component of the APR that facilitates pathogen opsonization, lysis, and the recruitment of additional immune cells, also appears to be active in bats exposed to *P. destructans* (41). Blood plasma from *M. lucifugus* hibernating in WNS-affected sites exhibited enhanced complement activation compared to plasma from bats in unaffected locations, and demonstrated increased bactericidal activity but a reduction in fungicidal capacity (41). These findings suggest that *P. destructans* may elicit complement-mediated immune responses, although the system may be primarily adapted to target bacterial pathogens, potentially limiting its effectiveness against fungal invaders (41). The observed reduction in antifungal activity remains poorly understood and the overall role of the complement system in the WNS immune response warrants further investigation. Combined data from studies on both North American and European bats reveal that WNS infection also leads to differential expression of numerous immune-moderating cytokines and chemokines, many of which point toward the initiation of an immune response (15, 29, 36, 38). For instance, annexin family proteins, such as ANXA6, are involved in membrane trafficking and repair, and regulate inflammatory reactions in many mammals, indicating cellular stress or damage in infected wing tissue (**Table 1**; 29, 42). Elevated iNOS2, an enzyme that catalyzes the production of nitric oxide, further signifies a reactive nitrogen species–mediated antifungal response (**Table 1**; 43). Likewise, increased levels of the chemoattractant proteins CCL2, CCL20, and CCR1, glycoprotein G-CSF, and interleukin (IL)-8 promote neutrophil and granulocyte recruitment through chemotaxis and bone marrow stimulation, demonstrating an attempt to recruit these cells to infection sites (**Table 1**; 44). Similarly, general APR-mediatory cytokines such as tumor necrosis factor (TNF)α, IL-1β, and IL-6 support this response through pro-inflammatory signalling and stimulation of additional APR proteins (**Table 1**; 45). Despite the upregulation of these molecules, there is a marked absence of immune cells at sites of fungal infection, suggesting that although antigen recognition is occurring, other factors are inhibiting the successful initiation of an immune response (46).

**Table 1:** Comparison of notable antifungal immune molecule expressions in North American *M. lucifugus* and European *M. myotis* bats.

Immune molecule	Expression in North American *M. lucifugus*	Timepoint(s) of detection	Expression in European *M. myotis*	Timepoint(s) of detection	References
IL-1β	Upregulated (Wing tissue) No change (Keratinocytes)	71–73 days post infection (p.i.); >13 weeks p.i.; 4–8 h p.i.	No change (Blood sample)	Late hibernation, 0.5–96 h p.i.	(6, 15, 29, 36)
IL-4	No change (Wing tissue) Downregulated (Blood plasma)	16 weeks p.i.; early-late hibernation	No data	N/A	(33, 37)
IL-6	Upregulated (Wing tissue; keratinocytes; lymph nodes)	71–73 days p.i.; >13 weeks p.i.; 16 weeks p.i.	No data	N/A	(6, 15, 29, 37)
IL-8	Upregulated (Keratinocytes)	4–8 h p.i.	No data	N/A	(6)
IL-10	Upregulated (Wing tissue; lungs)	71–73 days p.i.; 120 days p.i.	No data	N/A	(15, 38)
IL-17A	Upregulated (Lymph nodes)	16 weeks p.i.	No data	N/A	(37)
IL-17C	Upregulated (Wing tissue)	>13 weeks p.i.	No data	N/A	(29)
IL-18	No change (Keratinocytes)	4–8 h p.i.	No data	N/A	(6)
IL-20	Upregulated (Wing tissue)	>13 weeks p.i.	No data	N/A	(29)
IL23A	Upregulated (Wing tissue; lungs)	>13 weeks p.i.; 120 days p.i.	No data	N/A	(29, 38)
IL24	Upregulated (Wing tissue)	>13 weeks p.i.	No data	N/A	(29)
IL-27A	Upregulated (Wing tissue)	71–73 days p.i.	No data	N/A	(15)
IFNγ	No change (Non-vaccinated bats; wing tissue) Upregulated (Vaccinated bats; spleen and axillary lymph nodes)	16 weeks p.i. (Non-vaccinated bats) 126 days p.i. (Vaccinated bats)	No data	N/A	(37, 39)
TNFα	Upregulated (Lungs) No change (Keratinocytes)	120 days p.i.; 4–8 h p.i.	No change (Blood sample)	Late hibernation, 0.5–96 h p.i.	(6, 36, 38)
TGFβ	Upregulated (Wing tissue; keratinocytes)	4–8 h p.i.	No data	N/A	(6)
CCL2, CCL20	Upregulated (Wing tissue; keratinocytes)	>13 weeks p.i.; 4–8 h p.i.	No data	N/A	(6, 29)
CCR1	Upregulated (Wing tissue)	71–73 days p.i.	No data	N/A	(15)
APR proteins (ex. TF, Hp)	Differential expression (Blood sample)	Not known	Upregulated (Hibernating bats; blood sample) No change (Non-hibernating bats; blood sample)	0.5–96 h p.i. (Hibernating bats) 24–48 h p.i. (Non-hibernating bats)	(34, 36, 40)
iNOS2	No data	N/A	No change (Blood sample)	0.5–96 h p.i.	(36)
ANXA6	Upregulated (Wing tissue)	71–73 days p.i.	No data	N/A	(15)
Cathelicidin	Upregulated (Lungs)	120 days p.i.	No data	N/A	(38)

In a 2020 study, Hecht-Höger et al. measured APR proteins in both healthy and *P. destructans*-infected *M. myotis* and *M. lucifugus* bats to assess activation of the APR (40). Experimental challenges using zymosan, a yeast antigen derived from the cell walls of *Saccharomyces cerevisiae*, have been used to discern similar findings in European bats, yielding contrasting results (34, 36). Zymosan challenges commonly induce an APR in experimental models, although responses can vary (34, 47, 48). In a 2022 study by Seltmann et al., European *M. myotis* bats were equipped with temperature-sensitive radio transmitters and experimentally challenged with zymosan to measure skin temperature changes associated with an APR (34). No measurable response was observed within 48 hours post-infection, based on key indicators such as leukocyte profiles, fever, body mass changes, and haptoglobin (Hp) levels, a protein that binds to hemoglobin to prevent oxidative damage (34, 49–51). Hibernating individuals of the same species showed a pronounced APR following a zymosan challenge after five months of torpor, however, marked by elevated Hp levels without increased arousal frequency; an atypical response among hibernating mammals (**Table 1**; 36, 52, 53). This may indicate an adaptation in torpid bats to enhance innate immunity against fungal pathogens, likely driven by high-density roosting conditions that elevate transmission risk. Supporting this, zymosan-challenged *M. myotis* bats exhibited significantly higher reactive oxygen metabolite levels than those challenged with a viral antigen or unchallenged controls, reflecting increased oxidative stress and immune activation specific to fungal antigens (54). The comparatively lower response to viral antigens aligns with the known viral tolerance of bats, highlighting a stronger physiological cost associated with fungal infection (54). Importantly, *M. lucifugus* bats also displayed upregulation in genes associated with oxidative stress when infected with *P. destructans*, as well as differential expression of serotransferrin (TF), a protein functionally similar to Hp (**Figure 1**; **Table 1**; 40). Elevated APR protein levels in these bats may buffer against oxidative stress during fungal infection, and appears to function in both European and North American bats. Notably, however, a 2013 study by Moore et al. found a reduction in total circulating antioxidants in bats from locations affected by WNS, theorizing that increased bouts of arousal may result in reductions in available antioxidants as pathologically-induced free radicals, such as reactive oxygen or nitrogen species, are neutralized (33). This strategy may be advantageous in early stages of hibernation, but likely diminish in efficacy as the production of free radicals outpaces that of the antioxidants (33). Species-specific studies across North American populations would help discern if increased levels of APR proteins and antioxidants have a greater capacity to mitigate WNS pathology in more resilient species.

## Adaptive response

### Antigen presentation by MHC molecules

In most mammalian infections, major histocompatibility complex (MHC) receptors present fragments of pathogenic proteins which then activate T-cells and B-cells to initiate an adaptive immune response (55). In humans and other mammals, MHC molecules are categorized as class I and class II, which primarily present intracellular pathogens, such as viruses, and extracellular pathogens, such as fungi, respectively (55, 56). Cross-presentation may also occur to ensure controlled regulation of immune responses (57). Bats possess both canonical subsets of MHC molecules, with MHC class II likely responsible for recognition of *P. destructans* in the host, although cross-presentation of exogenous fungal fragments on MHC class I molecules has not been investigated and should not be discounted (Colbert et al., 2021; 58–60). MHC genes in many vertebrates exhibit high polymorphism, enabling diverse immune responses to pathogens (61–64). This high polymorphism is also observed in several bat species, likely as an evolutionary adaptation of exposure to a wide range of pathogens (65–67). A 2020 study by Yi et al. examined MHC class II isotype DR-β (MHC II *DRB*) gene variation across *M. lucifugus* populations with differing histories of *P. destructans* exposure, comparing pre- and post-WNS infection groups (60). Analysis of wing tissue samples revealed no significant genetic differentiation in MHC class II genes associated with WNS, suggesting MHC polymorphism is not a primary factor in the survival of North American bats post-infection. Similar studies should be conducted across additional populations of *M. lucifugus* as well as other species impacted by WNS to validate this hypothesis. Nevertheless, MHC class II genes likely play a key role in recognizing *P. destructans* and activating T-cell-mediated fungal clearance. Furthermore, previous studies in thirteen-lined ground squirrels suggest that antigen presentation in hibernating mammals is temperature dependent, with low body temperatures during torpor impairing the detection of pathogens (68). If a similar mechanism occurs in bats, it could explain the ability of *P. destructans* to persist on the cold skin of hibernating individuals without host detection, thereby delaying the initiation of an effective immune response.

### T-cell differentiation and activation

During late stages of infection, *P. destructans* fungal load increases, resulting in lesions on wing and tail membranes (5). By this time, T-cell differentiation and activation begins to occur. Compared to other mammals, bats display notably delayed T-cell activity, with current data suggesting a peak response attained after over 120 hours post-infection *in vitro*, compared to only 48 hours in mice and 72 to 96 hours in humans, allowing for additional fungal proliferation without an adequate host response (69, 70). Studies on Australian *Pteropus alecto* bats have found that this species possess the same primary and secondary lymphoid organs as other mammals, including the thymus, bone marrow, spleen, and lymph nodes, and share many typical T-cell subsets found in humans (59, 71–73). Although fruit bats such as *P. alecto* are somewhat evolutionarily distant from the species impacted by WNS, the physiological conservation of these features may assist in explaining the immune response to the disease (74). In wild-caught *P. alecto* bats, CD4^+^ T cells are primarily located in the lymph nodes and bone marrow, while CD8^+^ T cells are mostly found in the spleen, although both cell types are generally present throughout lymphoid organs at levels significantly higher than in humans and mice (72). Notably, the CD4^+^:CD8^+^ ratio in the bat bone marrow is approximately 2:1, contrasting with the 1:2 ratio typically seen in humans (72, 75). CD4^+^ T cells play a critical role in initiating and coordinating immune responses by activating B-cells, CD8^+^ T cells, macrophages, and dendritic cells to neutralize and clear infected cells (76). In this sense, assuming similar T-cell distributions and ratios in temperate bat species, this skew toward CD4^+^ T cells in bone marrow may facilitate rapid recruitment of immune cells to common fungal entry points such as epithelial tissues.

CD4^+^ T cells differentiate into effector subsets tailored to combat specific pathogens (77). In the context of white-nose syndrome, the T-helper (Th)17 subset of CD4^+^ T cells is generally thought to be important for defending against extracellular fungal pathogens such as *P. destructans* (**Figure 1**; 78). In humans, Th17 cells produce cytokines such as IL-17, IL-23 and IL-1β, which promote the recruitment of neutrophils, enhance epithelial barrier integrity, and stimulate the production of antimicrobial peptides (78–81). Furthermore, the observed down-regulation of IL-4 diminishes the likelihood that a Th2-mediated response is being initiated (33). Indeed, in naturally infected bats, the expression patterns of these molecules signifies a polarization towards a Th17 response similar to that of humans, although as described prior, early recruitment of immune cells is largely unsuccessful during the APR (**Figure 1**; 5). This discrepancy may be explained by the bouts of torpor and arousal during hibernation, in which each instance of arousal initiates a Th17 response that activates immune cells, although the short period may not provide adequate time for phagocytes and lymphoid cells to migrate to infected skin before the subsequent bout of torpor, rendering the attempted Th17 response ineffective (6, 82). Alternatively, or in concert with this, the impairment of immune cells may be a result of immune evasion or immunosuppressive strategies employed by *P. destructans*. For instance, elevated IL-10 expression, which is a cytokine known for its anti-inflammatory effects and ability to suppress antigen presentation, may contribute to delayed or insufficient adaptive immune responses (37, 83). Indeed, during the early stages of *P. destructans* infection, *M. lucifugus* bats exhibit elevated metabolic activity even before disruptions to torpor–arousal cycles occur, suggesting an increase in energy expenditure from infection and immune cell activation. (84).

**Figure 1**. Current known mechanisms involved in a white-nose syndrome infection in North American bats. *P. destructans* conidia enter via the skin. Early defense occurs with the cutaneous microbiome. Internalized antigen is recognized by TLR and CLR pattern recognition receptors, initiating a cascade of signalling molecules. Antimicrobial peptides, such as cathelicidin, are recruited to disrupt fungal growth. Preliminary recruitment of innate immune cells, including acute phase response (APR) proteins, macrophages, and neutrophils, is generally unsuccessful. Th17-type CD4^+^ T-cells induce additional cytokines which assist in further immune cell recruitment. Macrophages ingest the pathogen and present fragments for recognition by CD8^+^ T-cells and B-cells. CD8^+^ T-cells induce apoptosis in infected host cells. B-cells produce antibodies, which may undergo class-switching from low-affinity isotypes, such as IgM, to those with greater efficacy in antifungal defense, such as IgA or IgG. Antibodies support macrophages in antigen presentation, promoting clearance via phagocytosis and induced apoptosis.

### Th1/Th17 antagonistic WNS response

Th1 cells, which notably secrete IFNγ, play a central role in activating macrophages and promoting fungal clearance (85, 86). This pathway has also been implicated in bat antifungal immunity. In a vaccine study, Rocke et al. (39) administered a recombinant viral vector expressing *P. destructan*s antigens to *M. lucifugus* bats orally and through injection, in two separate trials (39). Following a *P. destructans* challenge, CD4^+^ T-cells from vaccinated bats produced elevated levels of IFNγ, indicative of a Th1-skewed immune response (39). In contrast the cytokine profiles of the Th17 activation in naturally infected wild bats, which display elevated transcript levels of IL-1β, IL-6, IL-23A, and IL-17C, expression of IL-17A was not significantly increased in vaccinated individuals, (29, 38, 39, 87). Vaccinated bats from both trials exhibited higher rates of survival compared to controls, with orally vaccinated bats, specifically, demonstrating higher mean weights at the time of death (39). These findings suggest that while Th17 signaling predominates during natural *P. destructans* infection, effective immune protection, such as that elicited through vaccination, relies more heavily on Th1-associated pathways. With this in mind, vaccination of entire bat populations may not represent a comprehensive solution for WNS prevention in North American species. Although vaccines may demonstrate efficacy under controlled experimental conditions, their deployment across wild populations presents significant logistical challenges, as bats frequently roost in inaccessible locations and move between sites, complicating efforts to track individual vaccination status (88). Moreover, vaccine-induced immunity is unlikely to be permanent, potentially waning over several years as *P. destructans* continues to evolve, leaving individuals susceptible unless booster administrations are feasible (88). Partial immunity, in particular, may limit the ability of a vaccine to fully prevent infection or transmission, resulting in heterogeneous outcomes across individuals and populations and complicating predictions about overall disease control (88). Nevertheless, vaccinating even a portion of a population could reduce overall fungal load, thereby mitigating disease spread without requiring universal coverage (88). While vaccination may appear to be a promising intervention, the practical complexities of administration currently limit its applicability, and fostering the natural development of resistance within North American bat populations may ultimately remain the most viable long-term strategy. Regardless, experimental vaccine studies continue to provide valuable insights into the interplay between Th1 and Th17 immune pathways in bats, offering a mechanistic understanding of why certain species may fail to mount effective responses against WNS.

Taken together, the current available evidence on the immunological response of bats indicates that WNS-susceptible bats may be functionally constrained within a Th17-dominant immune state, impairing the activation of Th1-mediated antifungal mechanisms required for fungal clearance (29, 37). This skewed immune polarization may reflect an evolutionary trade-off in bats, in which the immune system is adapted for viral tolerance through the suppression of excessive proinflammatory Th1-type responses, thus inadvertently hindering the effective mounting of Th1 responses against fungal pathogens. In addition, low-level constitutive expression of cytokines which support the Th17 state may further dampen Th1 polarization (72). Typically, the combined upregulation of IL-6 and transforming growth factor (TGF)β influences the differentiation of naïve CD4^+^ T-cells to a Th17 phenotype, and increased expression of IL-23 assists in the survival and maintenance of these Th17-type T-cells (89). Furthermore, the Th1 and Th17 pathways have several antagonistic properties, such as the reciprocal relationships between IL-17/IFNγ, and IL-23/IFNγ, preventing simultaneous differentiation into both effector types (89, 90). Interestingly, approximately 40% of splenic T cells in *P. alecto* constitutively express IL-17, IL-22, and TGFβ, which may suggest a natural skew towards a Th17 response. Coupled with the expression of IL-6, TGFβ, and IL-23 detected in WNS-positive North American bats, it is possible that the polarization towards a Th17 response is inhibiting the success of a necessary Th1 response. While such immune modulation may confer advantages in the virus-rich ecological contexts in which many bat species evolved, this same feature appears to be maladaptive for North American bats that encounter fungal pathogens, where robust Th1-mediated responses are required for effective clearance (32). In contrast, the lower susceptibility of European bat species is often attributed to long-term co-evolution with *P. destructans*, which has likely selected for immune strategies that permit fungal control and tolerance, such as a more effective Th1-skewed response. Likewise, various physiological and ecological factors may also contribute to this relationship, including variations in hibernacula selection, behaviours during hibernation, and inhibitory skin bacteria in comparison to North American counterparts (91–93). As a consequence, persistent Th17 signaling in North American bats may promote chronic inflammation and tissue pathology, contributing to the extensive wing damage and energetic depletion characteristic of WNS. This pattern parallels observations in human systemic lupus erythematosus in which elevated Th17 cell activity exacerbates disease and shifts immune responses toward a Th17-dominated profile, which introduces the possibility for anti-IL-17 interventions to promote a Th1-biased response in bats (94). Moreover, in other mammals, Th1 and Th17 responses often occur at distinct times or anatomical sites, highlighting the need for longitudinal studies profiling cytokine dynamics in WNS-infected bats across multiple time points (95).

CD8^+^ T cells also contribute to antifungal immunity, particularly during the later stages of infection. In humans and mice, these cells promote fungal clearance by inducing apoptosis in infected host cells (**Figure 1**; 96). The unusually high abundance of T-cells in bat bone marrow compared to humans and mice suggests a potential role for this tissue as a reservoir for adaptive immune memory (72). In humans and mice, CD8^+^ memory T-cells persist in bone marrow niches, allowing for efficient recall responses and enhanced immunosurveillance (97, 98). A similar mechanism may be operative in bats, with memory T-cells allowing for efficient clearance of reoccurring fungal infections (5). Specific surface markers for memory T-cells have yet to be identified in any bat species, largely due to a lack of cross-reactive antibodies for canonical markers such as the proteins CCR7, CD62L, and CD44, making the characterization of memory T-cells difficult (99). Furthermore, no surveys have been conducted on the reinfection rate of WNS in bats; that is, bats that were infected with *P. destructans*, overcame the infection, and were infected again a following year. Therefore, although the role of memory T-cells in persisting populations of bats facing a secondary WNS infection is unknown, this measurement may be useful in forecasting the long-term survival of vulnerable species considering the persistent nature of *P. destructans*. In addition to memory T-cells, CD8^+^ T-cells differentiate into functional subsets depending on the response type initiated (96). Th1-type CD8^+^ T cells exhibit cytotoxic activity necessary for pathogen clearance, whereas Th17-type CD8^+^ T cells recruit additional neutrophils to sites of infection, although excessive Th17 responses can lead to damaging inflammation, which may explain the increase in Th-17-associated cytokines and simultaneous progression in pathology in WNS-affected bats (96). Th17-like CD8^+^ T cells arise in the presence of IL-6 and TGF-β, and are likely involved in the polarization towards a Th17-type response in North American bats (96). Constitutive Th17-like expression in bat splenic cells supports the theory that when bats are challenged with a fungal antigen such as *P. destructans*, Th17-skewed CD8^+^ T cells may dominate, further promoting this deleterious pathway (72, 96, 100). This additional bias toward a Th17 response should be considered when evaluating the underlying drivers of Th17 activation during WNS.

### Antibody production

While the role of T-cells in bat antifungal immunity has yet to be clearly defined, current data underscores an importance for B-cells and antibodies as well. Previous work on antibody-mediated immune responses in bats collectively suggest that rather than creating highly specific antibodies, bats invest greater energy in constructing an expansive repertoire of lower-specificity antibodies to a wider array of target antigens (101–105). This implies poor adaptive refinement of *P. destructans*-specific antibodies, however, and a subsequently dampened antibody-mediated antifungal response. Indeed, the role of antibodies in WNS infections is particularly evident when contrasting the variable antibody-mediated responses between European and North American bat species (103, 104). Current research suggests that bats mount an antibody response against *P. destructans*, but the efficacy of the response varies by species and geographical origin (**Figure 1**; 103, 104). A 2015 study by Johnson et al. investigated several North American species known for WNS susceptibility, including *M. septentrionalis, P. subflavus, Corynorhinus rafinesquii, Nycticeius humeralis, Lasiurus borealis*, and *M. daubentoniid*, to determine the presence of antibodies to *P. destructans* (103). Upon collection, bats were visibly assessed for wing damage on site, and blood was collected to measure antibody tires using enzyme-linked immunosorbent assay (ELISA) (103). Populations of *M. lucifugus* bats in areas that had been to exposed to the fungus since 2006 and 2008, when it was first introduced in North America, displayed greater seroprevalence and titers of anti-*P. destructans* antibodies than naïve and more recently exposed populations, which also coincided with fewer skin lesions on the wing membranes of previously exposed bats (103). An antibody-mediated response may be favourable in populations with higher mean body sizes, where bats are able to expend more energy to disease clearance and reduced tissue damage. A 2009 paper by Reichard and Kunz stated that greater wing damage was associated with lower body mass, which was further supported by a 2015 paper by Johnson et al., which noted that bats in populations with higher antibody titres also had differences in behaviour and physiology that further assisted in their survival (103, 106). Thus, antibody-mediated immunity to *P. destructans* infections may be a favourable addition to immune response when energetically feasible, but is likely not the only factor determining survival.

Likewise, high antibody titres in European bats may correspond to less severe pathogenicity, although current research displays conflicting results on this (103, 104). The aforementioned study by Johnson et al. also collected samples from European *M. myotis* bats and found no detectable antibodies against *P. destructans* in both infected and uninfected individuals, suggesting that antibody-mediated immunity is not a primary mechanism for fungal resistance in these populations (103). In 2023, however, a contrary study by Pikula et al. found that increased production of *P. destructans* antibodies in two European bat species, *M. myotis* and *M. dasycneme*, may confer protection against the fungus, with bats possessing higher titres also displaying decreased damage to wing membranes, although the contradictory results may be a result of variations in sampling size and location and replicate studies are needed to confirm results (103, 104). North American bats, and likely European bats as well considering the relatively close phylogeny, express the five canonical heavy-chain antibody isotypes (107). Proteins mediating immunoglobulin class switching have been identified in several tropical bat species; however, this process has not yet been examined in bats infected with *P. destructans* (105). Investigating whether bats shift from predominant isotypes such as IgM to those more effective against fungal epithelial infections, including IgG or IgA, could provide valuable insight into adaptive immune responses during WNS (108). Although role of antibodies in antifungal immunity in bats may currently be unclear, the contrasting antibody profiles between European and North American bats suggest divergent immune strategies. North American bats may rely more heavily on cellular immunity, which is often compromised by hibernation, whereas European bats appear to employ balanced humoral and cellular responses enabling immune tolerance and fungal containment without excessive inflammation.

## Conclusions and future outlooks

Following a severe *P. destructans* infection in susceptible North American species, excessive energy expenditure from frequent arousals typically results in mortality from depleted fat reserves and subsequent emaciation (84). This outcome reflects a fundamental metabolic limitation of hibernation: thermoregulation during arousal, activation of immune pathways, and repair of damaged tissue each impose substantial energetic demands, and collectively exceed the narrow physiological budget available during torpor (84). Consequently, the reallocation of fat stores towards repeated arousal events and immunological processes associated with a *P. destructans* infection leaves inadequate reserves to sustain physiological stasis, thereby accelerating the dehydration, electrolyte imbalances, and physiological collapse characteristic of advanced WNS pathology (84). Interestingly, even upon overwinter survival of *P. destructans* infections, the sudden restoration of immune function in bats may result in immune reconstitution inflammatory syndrome (IRIS)-like dysregulation, causing continuous, often more severe pathology following arousal from hibernation (46). IRIS is characterized as a rapid worsening of pathological symptoms upon recovering from infection, an has been noted in immunocompromised humans challenged with bacterial or fungal infections (109). It is hypothesized that the stark contrast between immunosuppression during hibernation, allowing for extensive growth of *P. destructans*, followed by the subsequent euthermic state and restoration of metabolism results in uncontrolled inflammation, extreme tissue damage, and ultimate mortality (46). Although not well characterized, this post-emergence pathology may be the cause of considerable WNS-related deaths in North American bat populations (46). Assuming survival following infection and any subsequent post-infection, bats will shed remaining *P. destructans* hyphae along with any diseased or damaged cells, and form a new epithelium, returning to homeostatic conditions (5). By understanding the various mechanisms of WNS-induced mortality, specific measures can be strategically tailored to the tolerance and susceptibility of each species, enhancing the effectiveness of conservation efforts.

As a growing concern in modern ecological studies, climate change may present additional issues in understanding the dynamics of WNS by altering both the environmental suitability for *P. destructans* in hibernacula and the physiological state of bats that are exposed to the pathogen. As a psychrophilic fungus, the ideal temperature range for *P. destructans* growth is largely consistent with the current environments in which many bats roost over winter months (110). Variable ambient temperatures as a result of fluctuating climates may shift the seasonal time frame for fungal growth, potentially resulting in longer winters or infection periods that are incongruent to those of prior years, which may introduce novel method of exposure and ultimately exacerbate the rate at which bat populations encounter *P. destructans*. Concurrently, temperature exerts a strong influence on host immune function, with colder conditions suppressing adaptive responses and warmer conditions potentially disrupting the physiological cues that regulate torpor and arousal cycles (111). As a result, climate-driven variation in hibernacula conditions may shift the energetic and immunological trade-offs between torpor, thermoregulation, and immune defense, with important implications for WNS progression, fungal clearance, and overall bat survival. Collectively, there is no simple, comprehensive answer to clearing *P. destructans* in bats. Successful clearance appears to rely on a careful combination of several immunological factors including external skin environment, initiation of an effective APR involving innate immune cells, timely activation of T-cells and an energetically-efficient Th-type response, and if energetic demands permit it, secretion of antibodies. Nevertheless, many matters still remain unaddressed regarding the immune response of bats to a *P. destructans* infection. Further work is needed to understand the protective antifungal traits among North American bats, such as seasonal microbiome retention or genetic differences in immune regulation, contribute to varying levels of resistance and long-term survival. Considering the results of the study by Yi et al. mentioned prior, it would be valuable to investigate the expression of additional MHC class II and class I genes during *P. destructans* infection, to discern whether atypical antigen presentation or cross-presentation is occurring (60). Further investigation on the contradicting cytokine profiles of the Th1 and Th17 responses in North American bats will prove critical in the understanding of why North American bats succumb to WNS, with an emphasis on measuring these molecules at certain time points throughout hibernation periods, to note if the initiation of the Th17 response hinders any attempts to mount a Th1 response. Future studies using methods such as single-cell RNA sequencing should be conducted to detect the presence of novel cell types in bats, such as memory T-cell subsets. Surveys for WNS reinfection in both North American and European bats are also needed to gain insight on the persistence of the disease over seasons and if immune memory to this pathogen develops, ultimately helping to discern whether vaccination could be a potential strategy for long-term WNS mitigation (112). Notably, although zymosan is often used to model antifungal immune responses, it may not accurately reproduce the pathogenic and potential immunosuppressive mechanisms of *Pseudogymnoascus destructans* (6, 34). Furthermore, the metabolic and physiological disruptions exhibited in susceptible North American bats during WNS are challenging to study *in vivo* without worsening population declines. Therefore, *in vitro* models using cell lines from susceptible species such as *M. lucifugus* and *E. fuscus*, directly challenged with *P. destructans*, are needed (6, 34). Collectively, these studies provide insight on the host-pathogen dynamics between *P. destructans* and bats, and highlight multiple avenues for targeted therapeutic solutions to WNS in vulnerable populations. Continued research in these areas will reveal adaptations in the immunological responses that have emerged in North American bats and clarify the mechanisms underlying the greater resilience of European bats.
